# Curcumin-Based Inhibitors of Thrombosis and Cancer Metastasis Promoting Factor CLEC 2 from Traditional Medicinal Species *Curcuma longa*

**DOI:** 10.1155/2022/9344838

**Published:** 2022-01-17

**Authors:** Arun Chandra Manivannan, Minguel Carmena Bargueňo, Vinitha Devaraju, Punam Sen, Horacio Pérez-Sánchez, Abdul Kareem Mohammed Ghilan, Abdullah Farasani, Atif Abdulwahab A. Oyouni, Saad Ali S. Aljohani, Othman R. Alzahrani, Malik A. Altayar, Riyadh Hussain Sahal Aeban, Palanivel Velmurugan, Vinayagam Mohanavel, Thangavelu Sathiamoorthi, Ramaswamy Krishnaraj

**Affiliations:** ^1^Department of Microbiology, Science Campus, Alagappa University, Karaikudi 630003, Tamil Nadu, India; ^2^Universidad Católica de Murcia (UCAM), Guadalupe de Maciascoque, Murcia, Spain; ^3^Department of Microbiology, PSG College of Arts and Science, Civil Aerodrome Post, Coimbatore 641014, Tamil Nadu, India; ^4^Department of Botany and Microbiology, College of Science, King Saud University, P.O. Box 2455, Riyadh 11451, Saudi Arabia; ^5^Biomedical Research Unit, Medical Research Center, Jazan University, Jazan-45142, Saudi Arabia; ^6^Department of Medical Laboratory Technology, College of Applied Medical Sciences, Jazan University, Jazan-45142, Saudi Arabia; ^7^Department of Biology, Faculty of Sciences, University of Tabuk, Tabuk, Saudi Arabia; ^8^Genome and Biotechnology Unit, Faculty of Sciences, University of Tabuk, Tabuk, Saudi Arabia; ^9^Department of Basic Medical Sciences, Faculty of Medicine, Alrayan Colleges, Almadinah Almunawarah, Saudi Arabia; ^10^Department of Medical Laboratory Technology, Faculty of Applied Medical Sciences, University of Tabuk, Tabuk, Saudi Arabia; ^11^Comprehensive Specialized Clinics of Security Forces, Jeddah, Saudi Arabia; ^12^Consultant (Laboratory Medicine) Employment, Jeddah, Saudi Arabia; ^13^Centre for Materials Engineering and Regenerative Medicine, Bharath Institute of Higher Education and Research, Selaiyur, Chennai, Tamilnadu, India; ^14^Centre for Excellence-Indigenous Knowledge, Innovative Technology Transfer and Entrepreneurship and Department of Mechanical Engineering, College of Natural and Computational Science, Dambi Dollo University, Dambi Dollo, Ethiopia

## Abstract

The CLEC-2 receptor protein belongs to the C-type lectin superfamily of transmembrane receptors that have one or more C-type lectin-like domains. CLEC-2 is a physiological binding receptor of podoplanin (PDPN), which is expressed on specific tumour cell types and involved in tumour cell-induced platelet aggregation and tumour metastasis. CLEC-2 and podoplanin-expressing tumour cells interact to increase angiogenesis, tumour development, and metastasis. CLEC-2 is a hemi-immunoreceptor tyrosine-based activation motif (hemi-ITAM) receptor located on platelets and a subset of dendritic cells that are expressed constitutively. This molecule is secreted by activated platelets around tumours and has been shown to inhibit platelet aggregation and tumour metastasis in colon carcinoma by binding to the surface of tumour cells. Pharmacokinetic studies were carried using a DrugLiTo, and molecular docking was performed using AutoDock Tools 1.5.6 (ADT). Twenty-nine bioactive compounds were included in the study, and four of them, namely, piperine, dihydrocurcumin, bisdemethoxycurcumin, and demothoxycurcumin, showed potential antagonist properties against the target. The resultant best bioactive was compared with commercially available standard drugs. Further, validation of respective compounds with an intensive molecular dynamics simulation was performed using Schrödinger software. To the best of our knowledge, this is the first report on major bioactive found on clove as natural antagonists for CLEC-2 computationally. To further validate the bioactive and delimit the screening process of potential drugs against CLEC-2, in vitro and in vivo studies are needed to prove their efficacy.

## 1. Introduction

CLEC-2 (CLEC-1b) belongs to the C-type lectin superfamily of transmembrane receptors that have one or more C-type lectin-like domains (CTLDs) [1, 2]. C-type lectins are involved in a variety of processes including growth and development, respiration, blood coagulation, angiogenesis, and inflammation [3]. CLEC-2 controls a variety of physiological pathways by detecting and binding to endogenous and exogenous ligands [4]. While CLEC-2's participation in carcinogenesis and platelet activation is well-known, its role in thrombosis is unclear [5]. CLEC-2 has been linked to the inflammatory response, and overexpression of CLEC-2 ligands in inflamed tissues has been linked to vascular integrity, highlighting its function in thrombosis [6]. CLEC-2 and its ligands serve as a molecular link between platelets, immune cells, and target cells, as well as a unique mechanism for thrombosis. As a result, CLEC-2-related pathways could be used to treat thromboinflammation [7].

In human hepatocellular carcinoma, C-type lectin domain family 1 member B (CLEC1B) is a novel platelet-related molecule associated with TH (HCC). This molecule is secreted by activated platelets around tumours and has been shown to inhibit platelet aggregation and tumour metastasis in colon carcinoma by binding to the surface of tumour cells [8]. Although CLEC1B has recently been reported to be significantly downregulated in HCC tumours, the role of CLEC1B in HCC remains unclear. CLEC1B is a signature gene that has been linked to tumour progression [9]. CLEC1B contributes to platelet aggregation inhibition [10]. CLEC1B is significantly downregulated in HCC [9]. CLEC1B is involved in cancer metastasis [11, 12].

CLEC-2 has a molecular weight of 32 kDa and is found in significant concentrations on megakaryocytes and platelets [1, 2]. CLEC-2, formerly known as the snake venom protein receptor, functions similar to glycoprotein (GP) VI (GPVI) in activating Src (nonreceptor tyrosine kinase) or Syk (spleen associated tyrosine kinase) upstream of phospholipase C (PLC) 2 to trigger platelet aggregation [13]. It is mainly composed of an YXXL motif, two conserved serine sequences at locations 21 and 27, and a partially conserved threonine sequence at position 9, with the YXXL sequence necessary for signal transduction [14]. CLEC-2 binding to its cognate ligand causes tyrosine phosphorylation of one intracytoplasmic YXXL motif, which activates the semi-immunoreceptor tyrosine-based activation motif (ITAM) pathway downstream [15]. The semihelical long loop area of CLEC-2's binding surface is variable compared with other portions, and ligand interaction can move the cytoplasmic signal transduction domain of CLEC-2 closer together, increasing ligand-induced dimerization [16]. Based on its cytoplasmic tail phosphorylated peptide, CLEC-2 binds to the tandem SH2 domain of Syk in a 2 : 1 stoichiometry [17]. CLEC-2 is also expressed at relatively low levels on Kupffer cells, sinusoidal endothelial cells [18], dendritic cells, macrophages [13], B lymphocytes, and neutrophils generated during the inflammatory response, according to studies on transgenic mice [19]. Lowe et al. [20] established that CLEC-2 expression on neutrophils is most likely an off-target consequence of antibodies, and there are also assertions that CLEC-2 expression is most likely confined to mice [19], whereas macrophages express CLEC-2 after phagocytosing platelets. As a result, the distribution of CLEC-2 is not entirely understood [20].

CLEC-2 is a physiological binding receptor of podoplanin (PDPN), which is expressed on specific tumour cell types and involved in tumour cell-induced platelet aggregation and tumour metastasis [21]. CLEC-2 and podoplanin-expressing tumour cells interact to increase angiogenesis, tumour development, and metastasis [22]. Extensive thrombosis is seen in mice with lung tumours, which can be reduced by inhibiting CLEC-2. Furthermore, inhibiting CLEC-2 decreased plasma cytokine levels, alleviated cachexia, and extended the survival of tumour-bearing animals [23]. Researchers discovered that podoplanin was upregulated in the venous wall while trying to figure out the mechanism of cancer-mediated inflammation. CLEC-2 may thus have a role in tumour-induced thromboinflammation, and persistent long-term exposure to inflammatory cytokines causes thrombosis [7].

CLEC-2 is a hemi-immunoreceptor tyrosine-based activation motif (hemi-ITAM) receptor located on platelets and a subset of dendritic cells that are expressed constitutively. CLEC-2 activates platelets by interacting with its endogenous ligands podoplanin and heme [8]. Podoplanin is a transmembrane O-glycosylated mucin-type protein that is expressed on type I lung epithelial cells, fibroblastic reticular cells, lymphatic endothelial cells, and podocytes and is increased on inflammatory macrophages, TH17 cells, fibroblasts, and cancer cells [24]. Apart from its role in thrombosis, deletion of platelet-CLEC-2 or hematopoietic-podoplanin promotes cytokine storm and bacterial proliferation and dissemination during caecal ligation and puncture-mediated peritonitis [25]. It is unknown whether crosslinking podoplanin can influence macrophage phenotype, fate, or tissue inflammation. This is especially important in disorders characterized by platelet-bound podoplanin-positive macrophages, such as atherosclerosis, rheumatoid arthritis, and breast cancer [26].

Curcumin derivatives have a long history of constituting the Indian herbal medicinal library for many formulations, and modern-day science also proves the efficacy of curcumin plant-based derived compounds over a wide spectrum of diseases and infection both in vitro and in silico [27]. Cancer is found to contribute to the significant epigenetic modulator and to be specific inhibition of DNA methyltransferases (DNMTs), regulation of histone modifications via the regulation of histone acetyltransferases (HATs) and histone deacetylases (HDACs), regulation of microRNAs, and so on[28]. Curcumin is reported to induce apoptosis and proliferation inhibition of cancer cells as an act of anticancer treatment while suppressing a variety of cellular signalling pathways. Supplementary Figure S1 describes the details of curcumin compounds. A recorded investigation proves that curcumin expresses anticancer activity against breast cancer, lung cancer, head and neck squamous cell carcinoma, prostate cancer, and brain tumours [29]. Beyond cancer therapeutics, curcumin and its derivatives are an active chemical constituent, a class of polyphenols demonstrated in multiple chronic diseases: inflammation, liver disease, arthritis, neurodegenerative diseases metabolic syndrome, and obesity. The current work was carried to harness the therapeutic nature of cancer against CLEC 2 with anticipation to inhibit the thrombosis and cancer metastasis mediated by the corresponding protein.

## 2. Materials and Methods

### 2.1. Homology Modeling and Ramachandran Plot Analysis

Homology modeling was employed to construct the missing part in protein under consideration and was performed in the Phyre2 web tool [30]. Ramachandran plot analysis enables the visualization of the sterically allowed region in a protein, which is best exploited to indirectly validate the stereochemistry and stability of the protein. This was undertaken in the MolProbity web tool, hosted by Duke Biochemistry, Duke University School of Medicine [31].

### 2.2. Protein Preparation

The crystal structure of CLEC-2 (PDB ID: 3WSR) in a complex with O-glycosylated podoplanin was retrieved from RCSB Protein Data Bank. O-glycans in the structure enables stable expression on lymphatic endothelial cells for interaction with platelets [23]. The two chains are separately saved in PDB format, using Biovia drug discovery studio 2021 [32]. PDBQT conversion of the protein group after the addition of hydrogen and computing Gasteiger was performed by Autodock software. Now, the protein is ready for docking.

### 2.3. Binding Site Prediction

The ligand site bound to extracted protein was affirmed as the active site of 3WSR further was validated with active site prediction module of Biovia drug discovery studio 2021and CASTp web server, as there is no ligand in association with protein structure was identified [33]. The grid was placed using AutoDock tools software over the site.

### 2.4. Ligand Preparation

Data of active phytochemicals present in clove were acquired from Indian Medicinal Plants, Phytochemistry, and Therapeutics (IMPPAT), a curated database [34]. These structures subsequently were retrieved from ZINC database. For further exploitation of ligand, these were energy minimized and optimized using Avogadro software and saved as PDB. The retrieved 3D structures in the format of PDB were further used for the docking studies. The 2D structures of all ligands are listed in Supplementary Figure S2.

### 2.5. Molecular Docking

Molecular docking studies were carried out using AutoDockTools (ADT) (Scripps Research US) with the extension suite to the Python Molecular Viewer of MGL tools with Perl program. Energy minimization of protein was carried out in Swiss PDB viewer (SPDBV; aka, DeepView), while the ligand energy minimization was carried out in the Avogadro module [35]. Protein was processed by the deletion of water, addition of polar hydrogen, and merging of nonpolar hydrogen. Later, the Gasteiger and Kollman charges were added to ligand and protein before the preparation of the grid parameter file, respectively. The docking studies were performed using the Lamarckian genetic algorithm (LMA) and empirical free energy function with a standard protocol. The protein and ligand interactions were analyzed for various bondings like hydrogen, hydrophobic, 2D structure interaction in the Discovery Studio tool (Biovia, 2021 client) [36].

### 2.6. Pharmacokinetics Profiling

Adsorption, Distribution, Metabolism, and Excretion (ADME) analysis vouchsafes the pharmacokinetic properties that a ligand must boat to establish its function in the administered body. This property analysis was executed using DruLiTo software for ADME analysis [37]. ProTox is a web tool designed for in silico prediction of oral toxicities; it incorporates nearly 33 models to identify toxicity probability and can predict several possible toxic endpoints and target prediction.

### 2.7. Target Prediction

Target prediction studies compute the probable macromolecular target site of screened small molecules. This methodology aids to trace the bioactivity, side effects, and off-targets. Swiss Target Prediction tool gives a rough output of top targets that a compound might react into screened small molecules [38]. Targets identification is to identify potential protein targets that can lead to adverse reactions if the subjected lead for analysis binds to it during metabolism, and based on the results of target analysis, one can prioritize the substances for further intensive toxicological analysis. ProTox server was also employed to identify the major toxic routs it may undergo [39].

### 2.8. Molecular Dynamics Simulation

Based on the molecular interaction and binding score of the small molecule against the target molecule, the top-ranked complex molecules were selected for the molecular dynamics simulations studies. They were performed using the Desmond tool in the Maestro platform of the Schrodinger tool (Schrodinger Release, 2019). First, the complex molecule was refined by optimizing hydrogen bond and energy minimized using the OPLS3e force field; further, the complex molecule was solvated using the method of simple point charged (SPC) in the 3D orthorhombic box with a buffer distance of 10 Å. Finally, the whole system is designated for the simulation time of 100 ns with 1000 trajectory points under an NPT ensemble of constant pressure, temperature, and atom number.

## 3. Results

### 3.1. Homology Modeling and Ramachandran Plot Analysis

**Figure 1:**
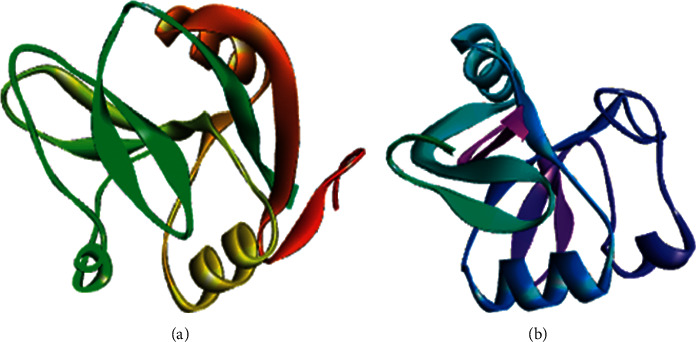
Representation of target protein (a) and (b) chain.

**Figure 2:**
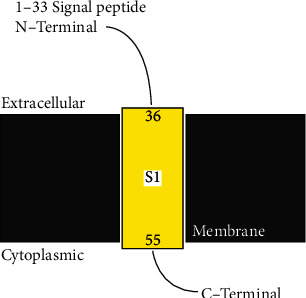
Membrane-spanning region of the protein.

**Figure 3:**
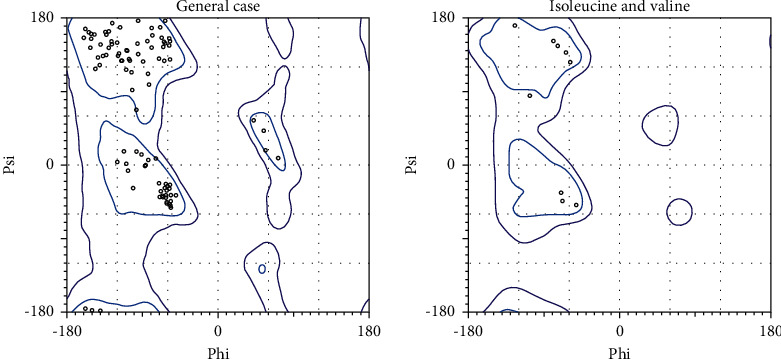
Ramachandran plot built by PolMorbity for chain A.

**Figure 4:**
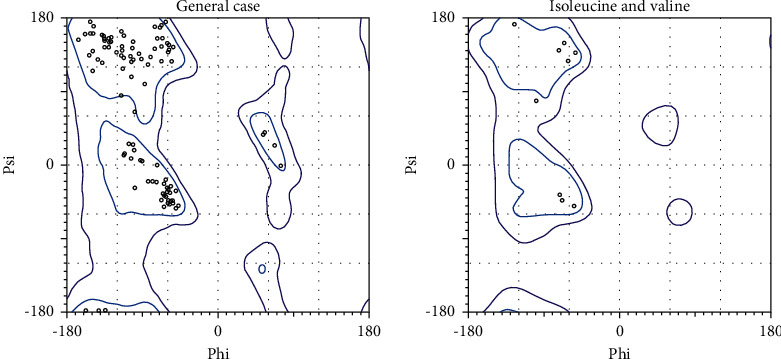
Ramachandran plot built by PolMorbity for chain B.

Homology modeling was performed for chain B of the protein as the construct was identified for missing residues in its chain (target protein, Figure 1(a) A chain, 1(b) B chain). The master input file was compared with the modeled pair and is found to have an RMSD value of 0.321 (Supplementary Figure S2 gives the superimposed visualization of modeled protein with native protein subset) and a TM-score of 0.91. RMSD indicates the root mean square distance between the set of pairs aligned while TM-score corresponds to a normalized score between the range 0 and 1, where 1 represents that they are identical; greater than 0.5, same overall fold; less than 0.2, no better than random. The modeled protein was subjected to further analysis. The pyre 2 web module also identified the membrane-spanning region(Figure S3) of the protein subunit that spans over residue 36 to 55 amino acid, and the region subjected for docking does not lie on this membrane-spanning region (Figure 2). Ramachandran plot aids to understand the fundamental structure of protein structure in terms of understanding the energetically allowed and disallowed region (Figures 3 and 4). The plot gives a 2D view of *φ*-*ψ* torsion angles prevailing in the backbone and aids to highlight the unrealistic conformations within the model. From the observation into 98.31% (minimum required Ramachandran favored >98%), residues of chain A fall into the category of highly favored as two of the residues, namely, Gly160 and 175, fall under the preferred region and none of the residues are questionable or unfavourable. Sometimes glycine being an exceptional amino acid, due to its achiral nature and very small structure deviation expressed by these amino acids, is acceptable. Possessing no more than one bad angle, no bad bonds, and a MolProbity score of 1.24 corresponding to 99th percentile, the protein chain subjected to Ramachandran plot analysis falls under the satirically preferred category. Supplementary Figures S3 and S4 correspond to Ramachandran plot analysis for chain A, while chain B with a MolProbity score of 0.78 lies over the 100th percentile, and except for three amino residues that lie under Ramachandran-favored region, all the residues remained over the allowed region (Supplementary Figures S4 and S5). llowed region. The protein was not recognized as having any bad angles or bonds, and Ramachandran distribution Z-score was in the range of −1.05 ± 0.66 (preferred limit: abs (Z-score) < (2)) [30].

### 3.2. Docking Studies

**Figure 5:**
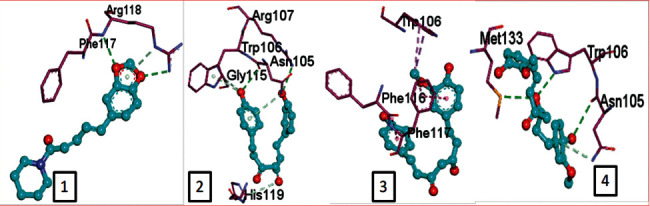
Hydrogen interaction by (a) piperine, (b) bisdemethoxycurcumin, (c) demethoxycurcumin, and (d) dihydrocurcumin.

**Figure 6:**
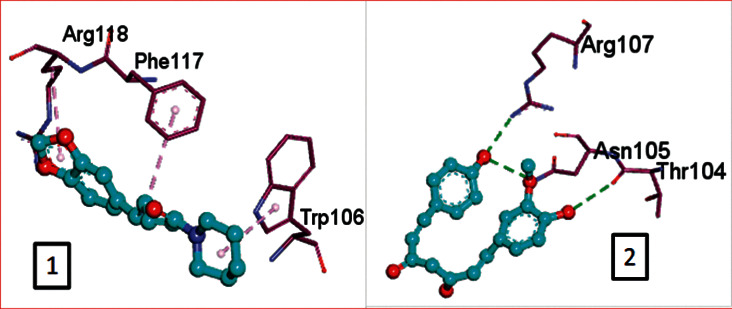
Hydrophobic interaction by (a) piperine and (b) demethoxycurcumin.

CLEC1B expression has recently been found to be substantially reduced in HCC tumours. Angiogenesis, tumour growth, and metastasis are all aided by CLEC-2 and podoplanin-expressing tumour cells interaction. In an experiment in mice with lung tumours, extensive thrombosis is found, which can be decreased by pronging CLEC-2 [6]. The CLEC-2 (PDB ID: 3WSR) in complex with O-glycosylated podoplanin was retrieved from RCSB Protein Data Ban; the structure constitutes two chains and while each chain interacting with their ligand molecules. Upon removal of ligands and followed by saving individual chains separately, docking was performed and the molecules exhibiting the best score were subjected for further analysis. Of 29 compounds, only four expressed a descent efficient binding score, while the rest falls in the range between -5 kcal/mole and −2.3 kcal/mol. The studies were taken forward with assuming these as hit compounds. Although other compounds like ascorbyl stearate, curcumin dimer 3, curcumin dimer 2, and curcumin dimer 1exhibited significant binding energy of −7 kcal/mol, −7.4 kcal/mol, −6.8 kcal/mol, and −7.6 kcal/mol respectively, their pharmacokinetic properties (Table 1) and violation from major Druglikenes rules including Lipinskis ROF restricted their further analysis. The docking study revealed that curcumin derivatives were more efficient in terms of binding energy score and four compounds, namely, piperine (−7.2 kcal/mol), dihydrocurcumin (−6.9 kcal/mol) interacting with B chain, bisdemethoxycurcumin (−9.0 kcal/mol), and demothoxycurcumin (−8.5 kcal/mol) interacting with A chain, are found to be the best compared with the rest of the compounds (Figure 5). Bisdemethoxycurcumin interacts with A chain with six hydrogen bonds webbed by five amino acid residues, namely, Arg107 (2.94 Å), Asn105 (2.98 Å, 3.09 Å), Trp106(3.60 Å), Gly115 (3.43 Å), and His 119 (3.74 Å), and does not exhibit hydrophobic interaction, while demethoxycurcumin, another efficient interactor of A chain, bonds with four hydrogens at Arg 107 (2.82 Å), Thr104 (3.40 Å), and Asn105 (3.40 Å, 3, 23 Å) positions and five hydrophobic interactions, Trp106 (3.93 Å, 4.91 Å), Phe117 (4.97 Å, 4.67 Å), and Phe116 (4.67 Å). The B chain interaction residue piperine interacts with Phe117 (3.06 Å) and Arg118 (2.90 Å and 3.05 Å) residues for hydrogen and Tro106 (4.58 Å), Phe117 (4.98 Å), and Arg118 (4.36 Å) for hydrophobic binding. Dihydrocurcumin with no hydrophobic interaction binds to protein only via hydrogen bond at the following positions Met133 (3.37 Å), Trp106 (2.92 Å), and Asn105 (3.36, 3.71 Å) (Figure 6).  Supplementary Fig. S2 comprises interacting residues of all the compounds subjected to docking studies (Table 2).

### 3.3. Pharmacokinetics Profiling

**Figure 7:**
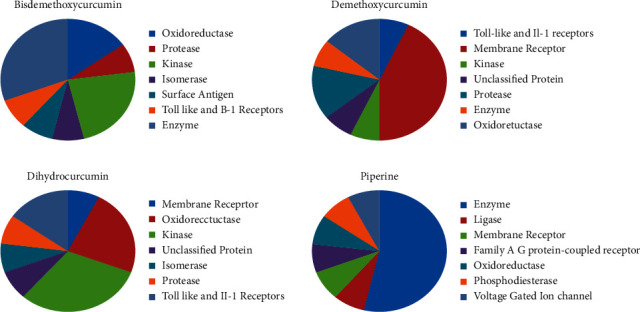
Predicted targets of selected top compounds.

Characterization for the lead likeness of ligand molecule was done by pharmacokinetics profiling ensuring that only the potential compound is subjected to further analysis. Pharmacokinetics property assigns the nature of druglikeness to hit compounds with some limitation accepted. A drug-like compound should have the following properties: molecular weight between160 and 480 g/mol, log P (logarithm of partition coefficient), or precisely lipophilicity within the range of −0.4 and 5.6. A negative log P indicates the compound is hydrophilic, 0 indicates that it is equally partitioned between lipid and aqueous phase, and a positive integer tags the lipophilic nature of the lead compound. Hydrogen bond acceptor (HBA) < 10, and hydrogen bond donor (HBD) < 5. TPSA corresponds to the total polar surface area and its value for an ideal drug is limited to below 140 Å. It has a corelation with molecular weight wherein when mass exceeding 500 TPSA lies beyond the range of 140 [30]. Atom molar refractivity (AMR) lies in the range 40–130; number of rotatable bond (nRB), ≤10; number of atoms, in the range between 20 and 70 numbers. Rotatable bond count (RC) lies in the range between 6 and 170. The number of rigid bonds (nRigidB), number of aromatic rings (nAromRing), and nHB are all factors determining the pharmacokinetics of a given compound [40]. For an ideal lead likeness, 250≤MW ≤ 350, XLOGP ≤3.5, and rotors ≤7. Bisdemethoxycurcumin and piperine both exhibit similar behavior; the former is moderately water-soluble while the latter is water-soluble significantly and both exhibit high GI absorption and BBB-permeable. Their log *K*_p_ (skin permeation) are −5.87 cm/s and −5.58 cm/s, respectively. They do not deviate from Lipinski, Ghose, Veber, and Egan's rules. They do not express any PAINS score and both express the bioavailability score of 0.55. Demethoxycurcumin and dihydrocurcumin are moderately water-soluble and have high GI absorption and are not BBB-permeable. Both of their log *K*_p_ (skin permeation) are −5.52 cm/s. A trace of violation from Lipinski, Ghose, Veber, and Egan's rules is found, and no PAINS alert is expressed. The bioavailability score for these two is 0.56. Ascorbylsterate violates Ghose druglikeness for atoms >70 and expresses 1 Brenk alert for having more than two esters. It also cannot be a lead-like molecule for the fact that its MW > 350, Rotors >7, and XLOGP3>3.5. Curcumin dimer 1 is poorly water-soluble, while having little or no GI absorption and being not BBB-permeable. It violates Lipinski Rule for MW > 500 and NorO >10 and deviates from Ghose rule for MW > 480, WLOGP >5.6, MR > 130, and no. of aTOMS >70. It also has MW > 350, Rotors >7, and XLOGP3 > 3. It cannot be categorized as lead-like molecule.  Supplementary Table S2 provides ADME characters of the next four best compounds that were excluded from further analysis for their violation from druglikeness character (Figure 7).

### 3.4. Target Prediction Studies

The results and interpretation of ProTox web server and Swiss target prediction for selective best compounds provide ideas to direct the intensive toxicological analysis. The toxic doses are counted as LD50 values using mg/kg of body weight as the unit of measurement, meaning that 50% of the subjects considered as test died upon exposure to the compound and were categorized into six classes: class I: fatal when swallowed (LD50 < 5); class II: same as class I (but has 5 < LD50 ≤ 50); class III: toxic if swallowed with an LD range greater than 50 and less than or equal to 300; class IV: harmful with LD50 greater than 300 and less than or equal to 2000; class V: harmful if swallowed (2000 < LD50 ≤ 5000); class VI: nontoxic with an LD > 5000. Bisdemethoxycurcumin is categorized as class V with an LD value of 2560 mg/kg and may interact with estrogen receptor ligand binding domain (ER-LBD) and mitochondrial membrane potential (MMP), which may correspond to a toxic interaction requiring significant attention, during the wet lab studies. Demethoxycurcumin with an LD50 value of 4000 is categorized as class V, toxic, and is predicted to elicit an immunotoxicity and active interaction with mitochondrial membrane protein (MMP) and a bit less probable interaction with heat shock response element (HSE) and nuclear factor (erythroid-derived 2)-like 2/antioxidant responsive element (nrf/ARE). The dihydrocurcumin is identified for LD5O value of 2000 mg/kg and thus is catergorized as class IV toxic substance, is assumed to trigger significant off-target interactions, including one that might result in immunotoxicity, actively interacts with peroxisome proliferator-activated receptor gamma (PPAR-gamma), nuclear factor (erythroid-derived 2)-like 2/antioxidant responsive element (nrf2/ARE), heat shock factor response element (HSE), and mitochondrial membrane potential (MMP), and may interact with phosphoprotein (tumour suppressor) p53. Piperine has the lowest predicted LD50 of all hovering around 330 mg/kg and is plugged over class IV toxic compounds. They have the potential to elicit immunotoxicity and less significant carcinogenicity. Their targets may include aryl hydrocarbon receptor (AhR), estrogen receptor alpha (ER), and ATPase family AAA domain-containing protein 5 (ATAD5).  Supplementary Tables S3–S6 provide top potential off-targets predicted for all four compounds.

### 3.5. MD Simulation Studies

The resulting top-ranked docking complex molecule was considered for performing MD simulation four complex molecules, which were passed from the simulation. In addition, the protein interaction with the ligand was studied throughout the total simulation time of 100 ns with 1000 projector points. Macromolecules and ligand cause interactions throughout the simulation time and are called contacts, classified based on hydrogen bonds, hydrophobic, ionic, and water bridges. The molecular dynamics simulation output was validated with an RMSD value around 3 Å distance and represented the stability of the complex molecule.

## 4. Discussion

**Figure 8:**
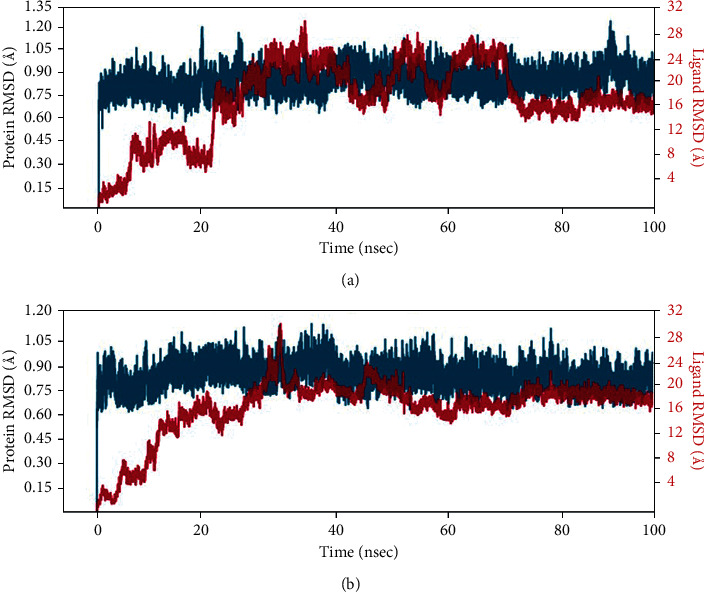
RMSD value of the complex molecule of (a) bisdemethocycurcumin and (b) demethoxycurcumin.

**Figure 9:**
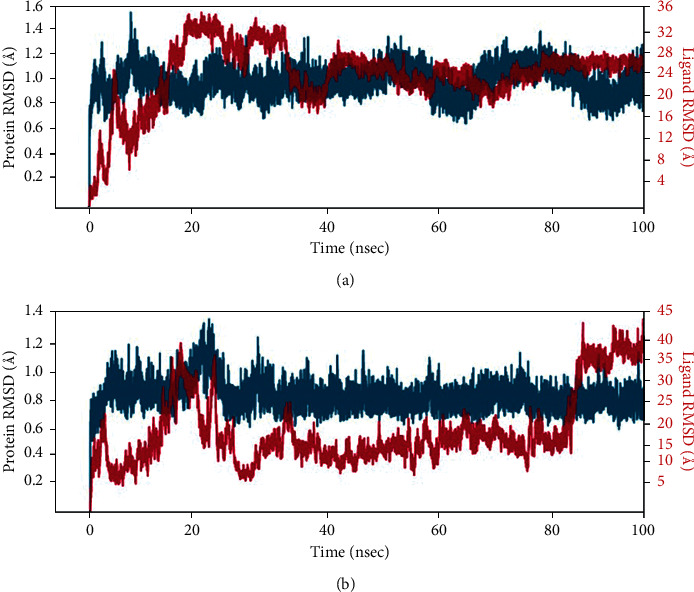
RMSD value of the complex molecule of (a) dihydrocurcumin and (b) piperine.

**Figure 10:**
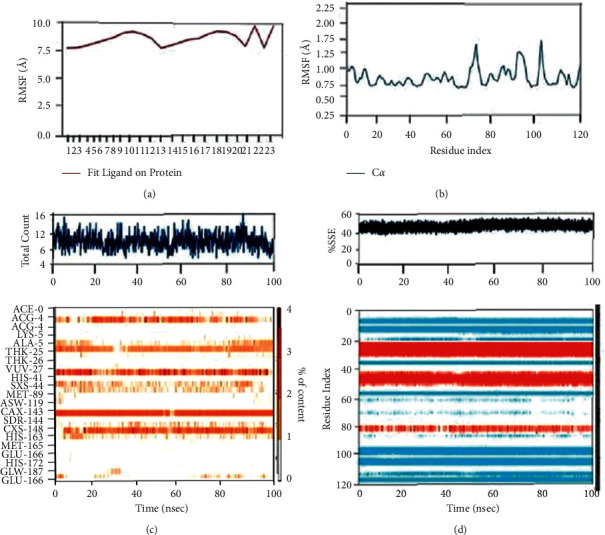
The complex molecule of bisdemethocycurcumin with the main protease of RMSF plot of protein and ligand (a, b), (c) protein-ligand contacts, and (d) protein secondary structure elements (SSE).

**Figure 11:**
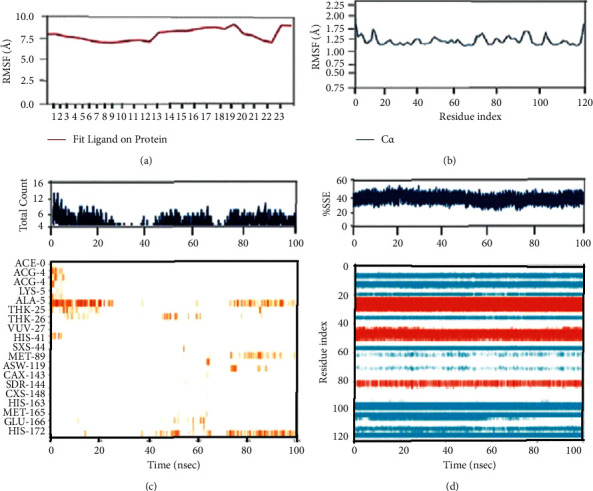
The complex molecule of demethoxycurcumin with the main protease of RMSF plot of protein and ligand (a, b), (c) protein-ligand contacts, and (d) protein secondary structure elements (SSE).

**Figure 12:**
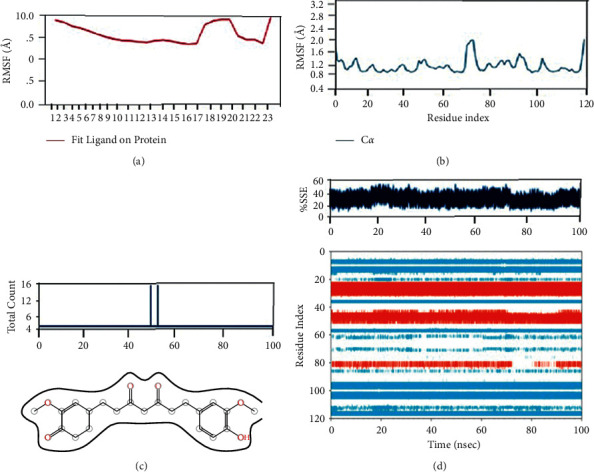
The complex molecule of dihydrocurcumin with the main protease of RMSF plot of protein and ligand (a, b), (c) protein-ligand contacts, and (d) protein secondary structure elements (SSE).

**Figure 13:**
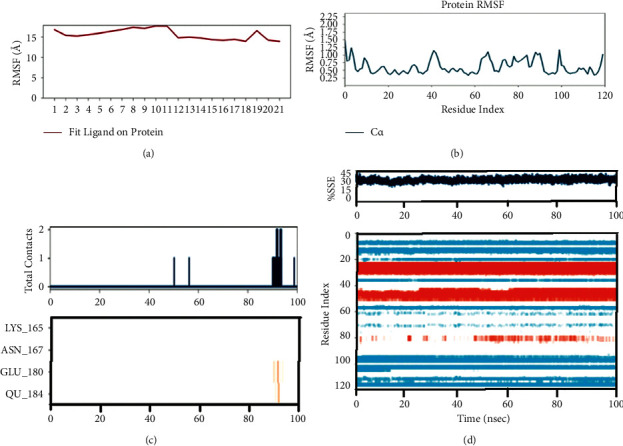
The complex molecule of piperine with the main protease of RMSF plot of protein and ligand (a, b), (c) protein-ligand contacts, and (d) protein secondary structure elements (SSE).

With the RMSD interpretation of bisdemethocycurcumin with the target protein, the complex molecules are shown stable around 45 ns after a significant fluctuation from time of placement in the alignment at 25 ns until 45 ns. Average deviation was around 1.9 Å and the small molecules tried to escape the cavity with the deviation of 1 Å distance, which holds the stability up to a total simulation time of 100 ns. On the other hand, the RMSD of demethoxycurcumin with target proteins, the complex molecules, deviates in the initial phase up from 16 Å to 28 Å and stabilizes with minimal deviation to 15 Å, which remains stable from 80 ns to 100 ns in around 1.8 Å RMSD deviation. RMSD results of bisdemethocycurcumin and demethoxycurcumin are graphed in Figures 8(a) and 8(b). Regarding the RMSD of dihydrocurcumin with target proteins, the complex molecules started to establish themselves only around 70 ns until which a clear fluctuation was witnessed around 2.1 Å deviation around the axis. Finally, piperine posed an oscillation and never settled over the template implying its randomness. The complex of all the molecules subjected for simulation studies originated in all proper binding poses with an acceptable RMSD value, and RMSD results of dihydrocurcumin and piperine are graphed in Figures 9(a) and 9(b). Regarding the bisdemethocycurcumin with the CLEC 2, the interaction showed discontinuous contacts in LYS150, ILE156, HIS199, and ASN210 and continuous contacts with Arg107, Gly115, and HIS154 across total simulation time. Demethoxycurcumin interaction showed discontinuous contacts in arg118 and TYR213 across total simulation time. On the other hand, the remaining two complex molecules dihydrocurcumin and piperine showed no significant contact (Figures 10–13). Unfortunately, dihydrocurcumin and piperine have no significant interaction under the simulation environment.

## 5. Conclusion

The computational approach for a screening of 29 phytochemical extracted from *Curcuma longa* a C type lectin-like receptor 2 CLEC-2 protein (PDB ID: 3WSR) enhances the cancer metastasis promoting factor by eliciting tumor cell-induced platelet aggregation. The validation of compounds by docking studies, ADMET screening, and target prediction was done before molecular dynamics simulation. The structure of the target proteins and small molecules were downloaded from the Protein Data Bank and ZINC database, respectively. The pharmacokinetic studies were evaluated for supporting ligand compound's characteristics, where top four compounds exhibited stronger drug-like character with no fluctuation from the rule of five and rule of three. These are piperine (−7.2 kcal/mol) and dihydrocurcumin (−6.9 kcal/mol) interacting with B chain and bisdemethoxycurcumin (−9.0 kcal/mol) and demothoxycurcumin (−8.5 *k* cal/mol). The scores represent their docking score interacting with chain A, and they were identified as a potential antagonist against target molecule. To further validate the top-ranked compounds with target proteins, these complexes were carried to MD simulation. From the simulation studies, the selected complex molecules revealed the stronger interaction with higher stability throughout the total simulation time. None of the compounds expressed any toxic characters and had no dreadful off-target interactions. The simulation studies revealed that only bisdemethocycurcumin and demethoxycurcumin exhibit better interaction under the simulation environment, while the remaining two do not show up any significant interaction. The interaction with chain B was not so significant and impactful; hence, these compounds were dully restricted from entering the simulation studies. These two complex molecules should further validate with in vitro and in vivo clinical phase studies.

## Figures and Tables

**Table 1:** Pharmacokinetic properties.

Properties	Bisdemethoxycurcumin	Demethoxycurcumin	Dihydrocurcumin	Piperine
MW	291.0	319.97	347.97	256.99
Log P	1.887	2.024	1.694	2.518
Log P	1.519	1.154	0.258	−0.635
HBA	4	5	6	4
HBD	0	0	0	0
TPSA	34.14	26.3	52.6	38.77
AMR	98.41	107.08	110.87	82.03
nRB	6	6	9	4
nAtom	23	25	27	21
RC	2	2	2	3
nRigidB	18	20	19	19
nAromRing	2	2	2	1
Nhb	4	5	6	4

**Table 2:** The interacting residues and their corresponding chain.

Name of ligand	Chain (A/B)	Interactions	
		Hydrogen	Hydrophobic
15 piperine 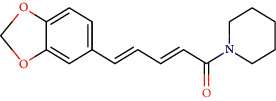	B	Phe117 (3.06 Å), Arg118 (2.90 Å and 3.05 Å)	Tro106 (4.58 Å), Phe117 (4.98 Å) and Arg118 (4.36 Å)
8 bisdemethoxycurcumin 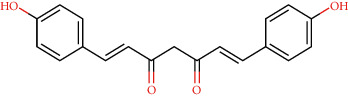	A	Arg107 (2.94 Å), Asn105 (2.98 Å, 3.09 Å), Trp106 (3.60 Å), Gly115 (3.43 Å) and His119 (3.74 Å)	—
11 demethoxycurcumin 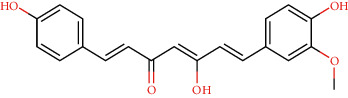	A	Arg 107 (2.82 Å), Thr104 (3.40 Å), and Asn105 (3.40 Å, 3,23 Å)	Trp106 (3.93 Å, 4.91 Å), Phe117 (4.97 Å, 4.67 Å), Phe116 (4.67 Å)
12 dihydrocurcumin 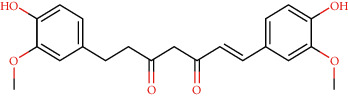	B	Met133 (3.37 Å), Trp106 (2.92 Å), Asn105 (3.36, 3.71 Å)	—

## Data Availability

The data used to support the findings of this study are included in the article. Further data or information required are available from the corresponding author upon request.

## Supplementary Materials

Supplementary Materials Supplementary Table S1: the details of curcumin compounds. Supplementary Table S2: pharmacokinetics. Supplementary Table S3: predicted targets of bisdemethoxycurcumin. Supplementary Table S4: predicted targets of demethoxycurcumin. Supplementary Table S5: predicted targets of dihydrocurcumin. Supplementary Table S6: piperine. Graphical Abstract: Supplementary Figure S1: superimposed image of modeled protein B chain (pink) and native structure (green). Supplementary Figure S2: Ramachandran plot for preproline and glycine of chain A. Supplementary Figure S3: Ramachandran plot for transproline and cisproline of chain A. Supplementary Figure S4: Ramachandran plot for preproline and glycine of chain B. Supplementary Figure S5: Ramachandran plot for transproline and cisproline of chain B.
