# Supplementary material for: Curcumin-Based Inhibitors of Thrombosis and Cancer Metastasis Promoting Factor CLEC 2 from Traditional Medicinal Species Curcuma longa
Source: Evid Based Complement Alternat Med. 2022 Jan 17;2022:9344838. doi: 10.1155/2022/9344838 (PMC8786508; doi:10.1155/2022/9344838)
Supplement: Supplementary Materials — Supplementary Table S1: the details of curcumin compounds. Supplementary Table S2: pharmacokinetics. Supplementary Table S3: predicted targets of bisdemethoxycurcumin. Supplementary Table S4: predicted targets of demethoxycurcumin. Supplementary Table S5: predicted targets of dihydrocurcumin. Supplementary Table S6: piperine. Graphical Abstract: Supplementary Figure S1: superimposed image of modeled protein B chain (pink) and native structure (green). Supplementary Figure S2: Ramachandran plot for preproline and glycine of chain A. Supplementary Figure S3: Ramachandran plot for transproline and cisproline of chain A. Supplementary Figure S4: Ramachandran plot for preproline and glycine of chain B. Supplementary Figure S5: Ramachandran plot for transproline and cisproline of chain B. [file 9344838.f1.docx]

| **S. No**  **Table S1: Describes the details of curcumin compounds** | **Name** | **Zinc ID** | **Chain** | **Interaction** | |
| --- | --- | --- | --- | --- | --- |
|  |  |  |  | **Hydrogen** | **Hydrophobic** |
|  | 1(1)Curcumin  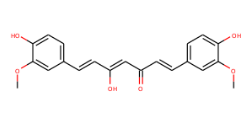 | ZINC100067274 | A | Trp106(2.96 Å), Arg118(2.89 Å, 3.08 Å) Phe117(3.10 Å) | Arg118(4.36 Å), Phe117(5.21 Å), Trp106(4.60 Å) |
|  | 1(2)Turmeric  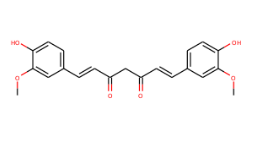 | ZINC899824 | A | Arg107(3.13 Å), Asn105(2.89 Å), Phe116(3.13 Å) | - |
|  | 1(3)1,9 Bis(4-hydroxyphenyl)-2,7-Nonadiene-4,6-Dione  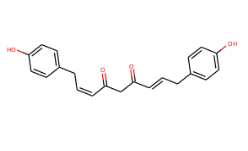 | Zinc14948330 | B | Arg107(3.00 Å), Phe116(3.20 Å), Phe117(3.25 Å) | - |
|  | 1(4) Curcumin li  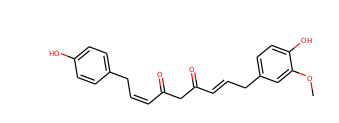 | ZINC16527488 | B | Arg107(2.81 Å), Asn105(3.33 Å), Trp106(3.22 Å, 3.33 Å), | Trp106(5.15 Å, 4.24 Å), Phe117(4.34 Å, 5.02 Å) |
|  | 1(5) Curcumin Pyrazole  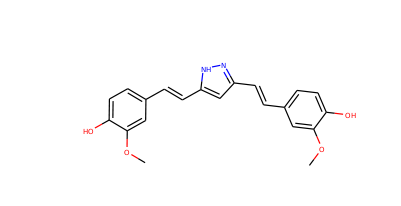 | ZINCE19816066 | B | Arg118(2.99 Å, 3.10 Å), Asp103(3.31 Å), Arg218(3.23 Å, 3.01 Å) | Trp106(5.09 Å), Phe117(5.41 Å) |
|  | 1(6) Curcumin I  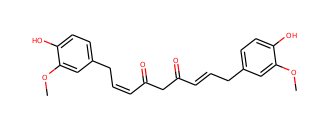 | ZINC85926636 | A | Arg118(3.13 Å, 3.13 Å), Trp106(3.13 Å) | His119(4.86 Å) |
|  | 1(7) Curcumin Dimer 1  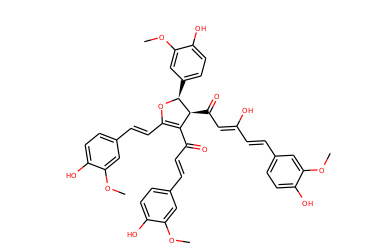 | ZINC150366575 | B | Asn120(2.80 Å, 3.06 Å), Arg118(3.00 Å), Phe116(3.07 Å), Tyr129(3.00), | Tyr213(5.57 Å), Met133(5.41 Å), Arg118(4.86 Å), His119(5.24 Å) |
|  | 1(11) Curcumin Dimer 2  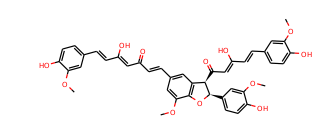 | ZINC150368101 | B | Asp103(3.35 Å), Arg218(2.95 Å), Arg118(3.05) | His119(5.42 Å), Met133(5.41 Å), Trp106(4.86 Å) |
|  | 1(16) Curcumin Dimer 3  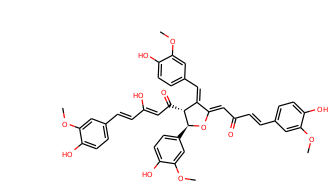 | ZINC150368132 | B | Trp106(2.98 Å, 3.22 Å), Met133(3.74 Å), Phe117(3.05 Å, 3.12 Å), Arg118(3.13 Å, 3.01 Å) | Trp106(4.21 Å), Met133(3.86 Å), Phe117(4.08 Å), Arg118(4.43 Å) |
|  | 2) [Alpha-Phellandrene](https://pubchem.ncbi.nlm.nih.gov/compound/442482)  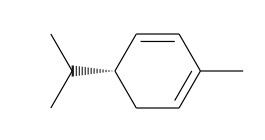 | ZINC8234325 | B | - | Phe117(5.11 Å, 5.28 Å), Met133(4.42 Å), Trp106(5.45 Å) |
|  | 3) Sabinene  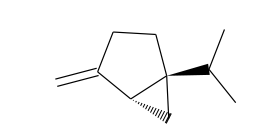 | ZINC1599725 | A | - | Arg118(5.22 Å) |
|  | 4) Demethoxycurcumin  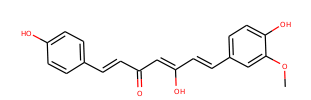 | ZINC100191509 | A | Arg118(3.39 Å, 2.83 Å), Trp106(3.18 Å), | - |
|  | 5) 2-Methylisoborneol  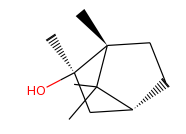 | ZINC2382779 | A | - | His119(4.06 Å), Arg118(4.05 Å) |
|  | 6) 2,6-Di-Tert-Butyl-4-Methylphenol  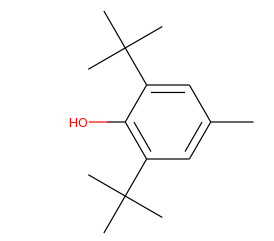 | ZINC1481993 | B | - | Phe117(5.36 Å, 4.32 Å), Trp106(3.92 Å, 4.85 Å), |
|  | 7) Ar-Tumerone  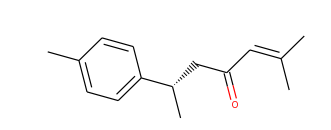 | ZINC6071066 | B | Arg118(3.07 Å, 3.26 Å) | Trp106(4.76 Å), Phe117 (5.39 Å), His119(4.95 Å) |
|  | 8) Bisdemethocycurcumin 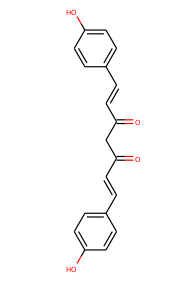 | ZINC1651126 | A | Arg107(2.94 Å), Asn105(2.98 Å, 3.09 Å), Trp106(3.60 Å), Gly115(3.43 Å) and His119(3.74 Å) | - |
|  | 9) 3-Tert-Buyl-4-Hydroxyanisole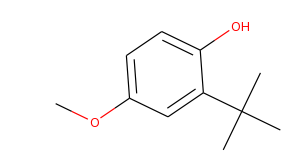 | ZINC1080 | A | Arg118(3.03Å, 3.26 Å), Asn105(3.04 Å) | - |
|  | 10(1) Curlone  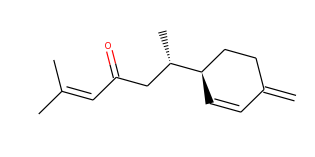 | ZINC365382 | B | Asn105(3.08 Å) | Trp106(4.79 Å), Arg118(4.19 Å), Phe117(5.29 Å, 5.27 Å, 5.15 Å, 4.48 Å), Met133(4.48 Å), Trp106(4.79 Å) |
|  | 11) Demethoxycurcumin  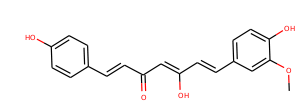 | ZINC100191509 | A | Arg 107(2.82 Å ), Thr104(3.40 Å), and Asn105(3.40 Å, 3,23 Å) | Trp106(3.93 Å, 4.91 Å), Phe117(4.97 Å, 4.67 Å), Phe116(4.67 Å) |
|  | 12) Dihydrocurcumin  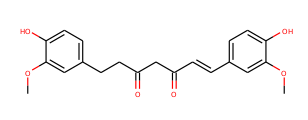 | ZINC13412576 | B | Met133(3.37 Å), Trp106(2.92 Å), Asn105(3.36, 3.71 Å) | - |
|  | 13) Eucalyptol  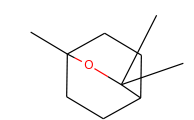 | ZINC967566 | - | Thr104(3.40 | - |
|  | 14(1) Ascorbic Acid  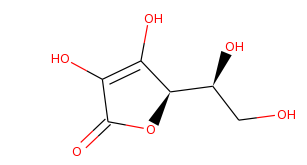 | ZINC100006770 | A | Arg118(2.82 Å, 2.94 Å, 3.02 Å), His119(3.27 Å), Arg157(3.38 Å, 3.17 Å) | - |
|  | 14(2) Ascorbyl Palmitate  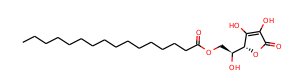 | ZINC100004322 | B | Arg218(2.97 Å, 3.16 Å), Trp106(2.90 Å, 2.80 Å), Asn105(3.16) | - |
|  | 15) Piperin  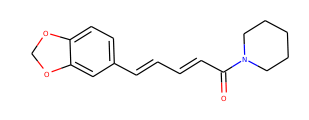 | ZINC1529772 | B | Phe117(3.06 Å) Arg118(2.90 Å) | Trp106(4.58 Å), Phe117(4.98 Å), Arg118(4.36 Å) |
|  | 16(1) dl-Alpha-Tocopheryl Acetate  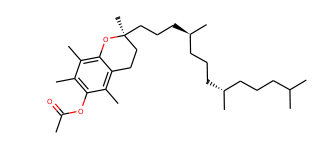 | ZINC3871495 | B | Trp106(2.20 Å) | Phe116(4.67 Å) |
|  | 16(2) Tocopherol  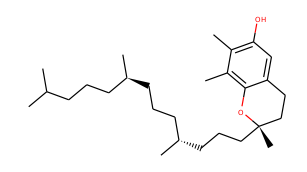 | ZINC2539618 | A | Arg118(4.92 Å), Phe117(5.04 Å), Trp106(5.02 Å), Met133(4.92 Å, 5.25 Å) | - |
|  | 16(6) Eprolin  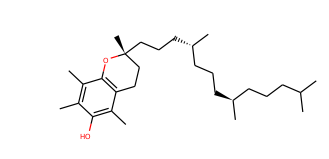 | ZINC4095858 | B | - | Phe183(3.83 Å, 4.11 Å, 5.12 Å, 5.03 Å), Val177(5.11 Å), Val169(5.41 Å), Trp170(5.41 Å) |
|  | 17(1) Escosyl  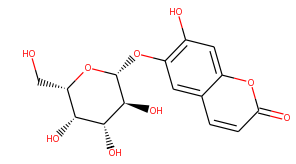 | ZINC19968 | A | Arg118(2.99 Å, 3.09 Å), Phe116(3.23 Å, 3.09 Å), Phe117(2.99 Å), Asn105(3.30), Trp106(3.10) | Phe116(5.22 Å) |
|  | 17(10) Ascorbyl Stearate  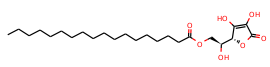 | ZINC100044734 | B | Trp106(2.89 Å, 3.01 Å), Arg218(3.04 Å, 3.25 Å, 3.10 Å), Asp103(3.10 Å) | Phe117(4.89 Å), Arg118(4.38 Å), His119(5.19 Å) |

Supplementary Table S1: Pharmacokinetics

| **Properties** | **Ascorbyl Stearate** | **Curcumin Dimer 1** | **Curcumin Dimer 2** | **Curcumin Dimer 3** |
| --- | --- | --- | --- | --- |
| **MW** | 399.96 | 695.94 | 695.9 | 695.9 |
| **Logp** | 9.35 | 3.371 | 3.371 | 3.371 |
| **Alogp** | -5.624 | 0.628 | 0.521 | 0.521 |
| **HBA** | 7 | 12 | 12 | 12 |
| **HBD** | 0 | 0 | 0 | 0 |
| **TPSA** | 52.6 | 80.29 | 80.29 | 80.29 |
| **AMR** | 94.31 | 222.43 | 222.35 | 222.35 |
| **nRB** | 20 | 14 | 14 | 13 |
| **nAtom** | 31 | 54 | 54 | 54 |
| **RC** | 1 | 5 | 5 | 5 |
| **nRigidB** | 11 | 44 | 44 | 45 |
| **nAromRing** | 0 | 4 | 4 | 4 |
| **nHB** | 7 | 12 | 12 | 12 |

Supplementary Table S2: Predicted targets of Bisdemethoxycurcumin:

| **Target** | **Common name** | **Uniprot ID** | **ChEMBL ID** | **Target Class** | **Probability** |
| --- | --- | --- | --- | --- | --- |
| Beta-secretase 1 | BACE1 | P56817 | CHEMBL4822 | Protease | 0.70988304 |
| Monoamine oxidase A | MAOA | P21397 | CHEMBL1951 | Oxidoreductase | 0.11827708 |
| Beta amyloid A4 protein | APP | P05067 | CHEMBL2487 | Membrane receptor | 0.11827708 |
| Histone acetyltransferase p300 | EP300 | Q09472 | CHEMBL3784 | Writer | 0.11827708 |
| Prostaglandin E synthase | PTGES | O14684 | CHEMBL5658 | Enzyme | 0.11827708 |
| Toll-like receptor (TLR7/TLR9) | TLR9 | Q9NR96 | CHEMBL5804 | Toll-like and Il-1 receptors | 0.11827708 |
| Coagulation factor VII/tissue factor | F3 | P13726 | CHEMBL4081 | Surface antigen | 0.11827708 |
| 11-beta-hydroxysteroid dehydrogenase 1 | HSD11B1 | P28845 | CHEMBL4235 | Enzyme | 0.11827708 |
| DNA topoisomerase II alpha | TOP2A | P11388 | CHEMBL1806 | Isomerase | 0.10161385 |
| Tyrosine-protein kinase SRC | SRC | P12931 | CHEMBL267 | Kinase | 0.10161385 |
| Glyoxalase I | GLO1 | Q04760 | CHEMBL2424 | Enzyme | 0.10161385 |

Supplementary Table S3: Predicted targets of Demethoxycurcumin

| **Target** | **Common name** | **Uniprot ID** | **ChEMBL ID** | **Target Class** | **Probability*** |
| --- | --- | --- | --- | --- | --- |
| Toll-like receptor (TLR7/TLR9) | TLR9 | Q9NR96 | CHEMBL5804 | Toll-like and Il-1 receptors | 0.967131413 |
| Epidermal growth factor receptor erbB1 | EGFR | P00533 | CHEMBL203 | Kinase | 0.599268558 |
| Carbonic anhydrase VII | CA7 | P43166 | CHEMBL2326 | Lyase | 0.574274832 |
| Carbonic anhydrase VI | CA6 | P23280 | CHEMBL3025 | Lyase | 0.574274832 |
| Carbonic anhydrase XII | CA12 | O43570 | CHEMBL3242 | Lyase | 0.574274832 |
| Carbonic anhydrase IX | CA9 | Q16790 | CHEMBL3594 | Lyase | 0.574274832 |
| Carbonic anhydrase XIII | CA13 | Q8N1Q1 | CHEMBL3912 | Lyase | 0.574274832 |
| Carbonic anhydrase VB | CA5B | Q9Y2D0 | CHEMBL3969 | Lyase | 0.574274832 |
| Beta amyloid A4 protein | APP | P05067 | CHEMBL2487 | Membrane receptor | 0.524103465 |
| Cyclooxygenase-1 | PTGS1 | P23219 | CHEMBL221 | Oxidoreductase | 0.515325374 |
| Arachidonate 15-lipoxygenase | ALOX15 | P16050 | CHEMBL2903 | Enzyme | 0.465502104 |
| Matrix metalloproteinase 9 | MMP9 | P14780 | CHEMBL321 | Protease | 0.448517242 |
| Beta-secretase 1 | BACE1 | P56817 | CHEMBL4822 | Protease | 0.448517242 |
| Nuclear factor erythroid 2-related factor 2 | NFE2L2 | Q16236 | CHEMBL1075094 | Unclassified protein | 0.431918667 |
| Arachidonate 5-lipoxygenase | ALOX5 | P09917 | CHEMBL215 | Oxidoreductase | 0.431918667 |
| 11-beta-hydroxysteroid dehydrogenase 1 | HSD11B1 | P28845 | CHEMBL4235 | Enzyme | 0.431918667 |
| Carbonic anhydrase I | CA1 | P00915 | CHEMBL261 | Lyase | 0.406896334 |
| Glycogen synthase kinase-3 beta | GSK3B | P49841 | CHEMBL262 | Kinase | 0.381806221 |
| Carbonic anhydrase IV | CA4 | P22748 | CHEMBL3729 | Lyase | 0.381806221 |
| Carbonic anhydrase II | CA2 | P00918 | CHEMBL205 | Lyase | 0.356609328 |
| Nitric oxide synthase, inducible | NOS2 | P35228 | CHEMBL4481 | Enzyme | 0.356609328 |
| Protein kinase C epsilon | PRKCE | Q02156 | CHEMBL3582 | Kinase | 0.339783364 |

Supplementary Table S4: Predicted targets of Dihydrocurcumin

| **Target** | **Common name** | **Uniprot ID** | **ChEMBL ID** | **Target Class** | **Probability*** |
| --- | --- | --- | --- | --- | --- |
| Arachidonate 5-lipoxygenase | ALOX5 | P09917 | CHEMBL215 | Oxidoreductase | 0.233302811 |
| Monoamine oxidase A | MAOA | P21397 | CHEMBL1951 | Oxidoreductase | 0.14525066 |
| Beta amyloid A4 protein | APP | P05067 | CHEMBL2487 | Membrane receptor | 0.14525066 |
| Histone acetyltransferase p300 | EP300 | Q09472 | CHEMBL3784 | Writer | 0.14525066 |
| Prostaglandin E synthase | PTGES | O14684 | CHEMBL5658 | Enzyme | 0.14525066 |
| Toll-like receptor (TLR7/TLR9) | TLR9 | Q9NR96 | CHEMBL5804 | Toll-like and Il-1 receptors | 0.14525066 |
| Beta-secretase 1 | BACE1 | P56817 | CHEMBL4822 | Protease | 0.121287003 |
| DNA topoisomerase II alpha | TOP2A | P11388 | CHEMBL1806 | Isomerase | 0.113285953 |
| Estradiol 17-beta-dehydrogenase 1 | HSD17B1 | P14061 | CHEMBL3181 | Enzyme | 0.113285953 |
| Nuclear factor erythroid 2-related factor 2 | NFE2L2 | Q16236 | CHEMBL1075094 | Unclassified protein | 0.113285953 |
| Cyclooxygenase-1 | PTGS1 | P23219 | CHEMBL221 | Oxidoreductase | 0.113285953 |
| Epidermal growth factor receptor erbB1 | EGFR | P00533 | CHEMBL203 | Kinase | 0.113285953 |
| Serine/threonine-protein kinase RAF | RAF1 | P04049 | CHEMBL1906 | Kinase | 0.113285953 |
| Serine/threonine-protein kinase B-raf | BRAF | P15056 | CHEMBL5145 | Kinase | 0.113285953 |
| Tyrosine-protein kinase SYK | SYK | P43405 | CHEMBL2599 | Kinase | 0.113285953 |
| Pyruvate dehydrogenase kinase isoform 1 | PDK1 | Q15118 | CHEMBL4766 | Kinase | 0.113285953 |
| Serine/threonine-protein kinase Chk1 | CHEK1 | O14757 | CHEMBL4630 | Kinase | 0.113285953 |
| Serine/threonine-protein kinase WEE1 | WEE1 | P30291 | CHEMBL5491 | Kinase | 0.113285953 |
| Tyrosine-protein kinase LCK | LCK | P06239 | CHEMBL258 | Kinase | 0.113285953 |
| Estradiol 17-beta-dehydrogenase 3 | HSD17B3 | P37058 | CHEMBL4234 | Enzyme | 0.113285953 |
| Glyoxalase I | GLO1 | Q04760 | CHEMBL2424 | Enzyme | 0.113285953 |

Supplementary Table S5: Piperin

| **Target** | **Common name** | **Uniprot ID** | **ChEMBL ID** | **Target Class** | **Probability*** |
| --- | --- | --- | --- | --- | --- |
| Monoamine oxidase B | MAOB | P27338 | CHEMBL2039 | Oxidoreductase | 1 |
| Sigma opioid receptor | SIGMAR1 | Q99720 | CHEMBL287 | Membrane receptor | 0.114337559 |
| Acetyl-CoA carboxylase 2 | ACACB | O00763 | CHEMBL4829 | Ligase | 0.097874534 |
| PI4-kinase beta subunit | PI4KB | Q9UBF8 | CHEMBL3268 | Enzyme | 0.097874534 |
| PI4-kinase alpha subunit | PI4KA | P42356 | CHEMBL3667 | Enzyme | 0.097874534 |
| Adenosine A2a receptor | ADORA2A | P29274 | CHEMBL251 | Family A G protein-coupled receptor | 0.097874534 |
| Steryl-sulfatase | STS | P08842 | CHEMBL3559 | Enzyme | 0.097874534 |
| Macrophage colony stimulating factor receptor | CSF1R | P07333 | CHEMBL1844 | Kinase | 0.097874534 |
| Anandamide amidohydrolase | FAAH | O00519 | CHEMBL2243 | Enzyme | 0.097874534 |
| 6-phosphofructo-2-kinase/fructose-2,6-bisphosphatase 3 | PFKFB3 | Q16875 | CHEMBL2331053 | Enzyme | 0.097874534 |
| N-acylsphingosine-amidohydrolase | NAAA | Q02083 | CHEMBL4349 | Enzyme | 0.097874534 |
| Acid ceramidase | ASAH1 | Q13510 | CHEMBL5463 | Enzyme | 0.097874534 |
| Transient receptor potential cation channel subfamily M member 8 (by homology) | TRPM8 | Q7Z2W7 | CHEMBL1075319 | Voltage-gated ion channel | 0.097874534 |
| Adenosine A3 receptor | ADORA3 | P0DMS8 | CHEMBL256 | Family A G protein-coupled receptor | 0.097874534 |
| Phosphodiesterase 4D | PDE4D | Q08499 | CHEMBL288 | Phosphodiesterase | 0.097874534 |
| Poly [ADP-ribose] polymerase-1 | PARP1 | P09874 | CHEMBL3105 | Enzyme | 0.097874534 |
| Cathepsin L | CTSL | P07711 | CHEMBL3837 | Protease | 0.097874534 |
| Cathepsin (B and K) | CTSB | P07858 | CHEMBL4072 | Protease | 0.097874534 |
| Diacylglycerol O-acyltransferase 1 | DGAT1 | O75907 | CHEMBL6009 | Enzyme | 0.097874534 |
| Serine/threonine-protein kinase Aurora-B | AURKB | Q96GD4 | CHEMBL2185 | Kinase | 0.097874534 |
| Cyclin-dependent kinase 1 | CDK1 | P06493 | CHEMBL308 | Kinase | 0.097874534 |
| Sodium channel protein type IX alpha subunit | SCN9A | Q15858 | CHEMBL4296 | Voltage-gated ion channel | 0.097874534 |
| Serine/threonine-protein kinase Aurora-A | AURKA | O14965 | CHEMBL4722 | Kinase | 0.097874534 |

**Graphical Abstract**

Figure S1: Superimposed image of Modelled protein B Chain (Pink) and native structure (green)

Supplementary Figure S2: Ramachandran Plot for pre-proline and Glycine of Chain A

Supplementary Figure S3: Ramachandran Plot for trans-proline and cis proline of Chain A

Supplementary Figure S4: Ramachandran Plot for pre-proline and Glycine of Chain B

Supplementary Figure S5: Ramachandran Plot for trans-proline and cis proline of Chain B
